# Nutritional Knowledge, Attitude, and Practices among Pregnant and Lactating Women Living with HIV in the Manzini Region of Swaziland

**Published:** 2014-06

**Authors:** Sakhile K.S. Masuku, Shu-Jan J. Lan

**Affiliations:** School of Nutrition and Health Sciences, College of Public Health and Nutrition, Taipei Medical University, No. 250 Wuxing St., Taipei 11031 Taiwan, ROC

**Keywords:** HIV/AIDS, Nutritional knowledge, attitude, practices, Pregnant and lactating women, Swaziland

## Abstract

The prevalence of HIV infection in Swaziland (26%) is among the highest in the world. We investigated nutritional knowledge, attitude, and practices (KAP) and the influence of sociodemographic factors on KAP among pregnant and lactating women living with HIV in the Manzini region of Swaziland. Interviews were conducted using a structured questionnaire to collect data from 324 subjects seeking healthcare from selected regional hospitals, health centres, and clinics in Manzini region. The results showed mean percentage scores of nutritional knowledge (67%), attitude (67%), and practices (51%) whereby educational level (p=0.002), employment status (p=0.009), income (p=0.008), religion (p=0.007), type of accommodation (p=0.006), type of transport used when going for shopping (p=0.001), and BMI (p=0.015) were significantly associated with nutritional practices. Significant positive correlations between nutritional KAP were observed: nutritional K and A (r=0.155, p=0.005), nutritional K and P (r=0.456, p=0.001), and nutritional A and P (r=0.230, p=0.001). Multiple linear regression analysis indicated that type of transport used when going for shopping (p=0.002), educational level (p=0.001), income (p=0.001), employment (p=0.038), knowledge of food proportion in a plate (p=0.000), a positive attitude towards high-fibre diet (p=0.004), and eating a variety of foods (p=0.006) were predictors of nutritional practices. Educational level was identified as a common predictor of nutritional knowledge, attitude, and practices, suggesting that both formal and informal education systems are potential factors influencing dietary practices among pregnant and lactating women living with HIV in Swaziland.

## INTRODUCTION

Human immunodeficiency virus (HIV) is a major global health problem. In 2011, it was estimated that 34 million people lived with HIV worldwide while 1.8 million deaths occurred (1). Sub-Saharan Africa accounts for 68% of people living with HIV globally (1). Swaziland ranks among countries with the highest HIV prevalence (26%). The impact of the epidemic has been adverse, resulting in low national life expectancy of 32 years (2). The prevalence of HIV is higher in women (31%) than men (20%). It rapidly increased from 3.9% in 1992 to 41% in 2010 among the pregnant women attending antenatal care (ANC) (3). Search results for HIV/AIDS in Swaziland from Wikipedia (the free encyclopedia) also provide information on prevalence, cultural background, national response, etc. (http://en.wikipedia.org/wiki/HIV/AIDS_in_Swaziland).

Nutritional alterations, such as weight loss and protein depletion are common in HIV infection, ultimately leading to malnutrition. On the other hand, poor nutrition results in a weakened immune system and, thus, predisposes an individual to opportunistic infections and enhanced progression of HIV to acquired immunodeficiency syndrome (AIDS) (4).

Experiences from different countries have shown that nutritional intervention is one of the key components of care and support for people living with HIV/AIDS (PLWHA), especially those in the developing world where access to antiretroviral drugs is limited (1). Maintaining good nutrition helps reinforce the effectiveness of the antiretroviral therapy (ART) (5). Several observational and randomized clinical trials suggested that micronutrient supplementation (vitamin A, B, C, and E), enhances the survival of HIV-infected individuals by mainly delaying the disease progression (6-9). According to Kaiser *et al.* (10), micronutrient supplementation significantly improved the CD4 cell count reconstitution in HIV-infected patients taking ART. Fawzi *et al.* (11) found that multivitamin supplementation during pregnancy and in the postpartum period resulted in significant improvements in haematologic status among HIV-infected women and their children. It has also been reported that low serum carotene concentration is common in AIDS patients and predicts death (12).

Nutritional knowledge and attitude are important factors of dietary practices and are, thus, potential targets for appropriate planning of nutrition care programmes for vulnerable people living with HIV and AIDS (PLWHA), such as pregnant women. Nutrition education enhances nutritional knowledge, thereby influencing attitude and practices towards good nutrition (13,14). However, sociodemographic factors have also been reported to affect the adoption of appropriate nutrition practices (15,16). In Swaziland, there is limited information on nutritional knowledge, attitude, and practices (KAP) among pregnant and lactating women living with HIV, and it is unclear to what extent the KAP are influenced by the sociodemographic factors. Therefore, the purpose of this study was to investigate nutritional KAP and determine their correlation with sociodemographic factors among pregnant and lactating women living with HIV in the Manzini region of Swaziland. Further, multiple regression sought to determine predictors of nutritional knowledge, attitude, and practices.

## MATERIALS AND METHODS

### Subjects and study design

A cross-sectional survey was conducted from July to September 2011 involving pregnant and lactating women living with HIV and receiving care from selected health facilities in the Manzini region of Swaziland. There are 4 regional hospitals, 1 health centre, and 9 clinics with maternal services in Manzini region. Two regional hospitals—Raleigh Fitkin Memorial (RFM) and Mankayane Government Hospitals)—were purposively selected. Random sampling was used in selecting a total of 4 clinics out of 9 clinics with maternal services. The estimation of sample-size was calculated using Raosoft software and was based on 95% confidence level, a margin error of 5%, and response distribution of 50%. The size of the sample yielded was 280 but, upon adjusting the size proportionately, a sample-size of 340 was used in the study. Participants were eligible for inclusion in the study if they were: HIV-infected pregnant and lactating women, aged 15-49 years, on ART or not, and pregnant or delivered in the selected health facilities. Medical charts were used for ascertaining HIV status of the subjects. Excluded cases were: HIV-positive pregnant and lactating women diagnosed with diabetes, vascular disease, heart disease, and kidney disease. Ultimately, data from 324 subjects were analyzed (10 subjects had missing data while 6 were not confirmed cases of HIV and, hence, excluded).

Ethical clearance was granted by the Scientific Ethics Committee (SEC), Ministry of Health, Swaziland, and informed written consent was obtained before each interview.

Data on nutritional knowledge, attitude, and practices of the respondents were obtained using a validated questionnaire developed by Action Against Hunger, a non-governmental organization in partnership with Swaziland National Nutrition Council. The questionnaire was slightly reviewed to include all indicators of interest and objectives for this study and was pretested among 20 HIV-positive pregnant and lactating women at Mbabane Government Hospital (n=10) and Siphocosini Clinic (n=10). The questionnaire was composed of four parts. The first part contained sociodemographic characteristics of subjects. The second part was composed of 12 nutritional knowledge-related questions with 2-point scale (0=wrong or ‘don't know’ response, 1=correct response). The knowledge scores were expressed as percentages of total points−12. The third part of the questionnaire included 12 items measuring attitude towards nutrition, using a 4-point Likert scale (0=strongly disagree, 1=disagree, 2=agree, and 3=strongly agree). To determine nutritional attitude of subjects, the negative phrases were recorded and allocated scores accordingly (0=most negative attitude, 3=most positive attitude). The attitude scores were expressed as percentages of total points−36. The fourth part of the questionnaire comprised items on consumption patterns in order to determine nutritional practices. Each question was scored using 2-point scale (1=most favourable nutritional practice, 0=undesirable nutritional practice). The practice scores were expressed as percentages of total points−41. The validity of the questionnaire was reviewed by two academics from Taipei Medical University and a nutritionist from Swaziland National Nutrition Council (reference no: MH/599C). A valid questionnaire with 68 variables consisting of 22 sociodemographic variables, 12 variables on nutritional knowledge, 12 variables on attitude and 22 variables on nutritional practice was eventually developed with Chronbach's alpha coefficient for knowledge (0.81), attitude (0.75), and practice (0.80) sections. The data were collected in a local language (Siswati) with the assistance of trained interviewers. In the current study, nutritional knowledge is hypothesized to interact with attitudes towards nutrition. Nutritional knowledge and attitudes, in turn, influence nutritional practices. Sociodemographic variables influence the nutritional KAP pathways.

### Statistical analyses

The SPSS (version 18.0) (SPSS Inc., Chicago, IL, USA) was used for data analyses. Data were presented as means±standard deviation (SD). Bivariate analyses (one-way ANOVA and independent *t*-test) were used in determining association between sociodemographic variables and nutritional KAP. The correlation between nutritional KAP and selected sociodemographic factors was assessed by the Pearson's product-moment correlation coefficient while multiple linear regression analysis was done to determine predictors of nutritional practices. The significance level considered was set at p<0.05.

## RESULTS

### Sociodemographic characteristics of participants

Out of the 340 respondents recruited, 10 (2.9%) had missing data, and 6 (1.7%) respondents were not confirmed cases of HIV and, hence, were excluded. Consequently, 324 respondents were considered for analysis. As shown in Table 1, the mean age of the participants was 27±6.1 years. Majority of the respondents (62%, n=201) had secondary education while 60% (n=195) were unemployed, 82% (n=267) used public transport when going for shopping, 70% (n=228) reported emotional ill-feeling for at least two weeks, and 41% (n=135) were obese (defined as BMI ≥30 kg/m^2^).

**Table 1.** Sociodemographic characteristics of pregnant and lactating women living with HIV in Manzini region (N=324)

Variable	n	%
Age (completed years)		
<20	37	11.4
20-24	87	26.9
25-29	96	29.6
30-34	56	17.3
≥35	48	14.8
Educational level		
None	20	6.2
Primary	73	22.5
Secondary	201	62.0
Tertiary	27	8.3
Informal (Sebenta)	3	0.9
Monthly income in Emalangeni*		
None	80	24.7
<500	62	19.1
500-1,000	69	21.3
1,001-3,000	95	29.3
3,001 and above	18	5.6
Marital status		
Single	157	48.5
Married	132	40.7
Cohabiting	26	8.0
Other (separated, divorced, widowed)	9	2.8
Religious group		
Christian	319	98.5
Other (Muslim, Traditional)	5	1.5
Employment status
Unemployed	195	60.2
Employed	106	32.7
Self-employed	23	7.1
Transport used when shopping		
Never go for shopping	8	2.5
Walk	21	6.5
Public transport	267	82.4
Car	28	8.6
Type of accommodation		
Own with mortgage	23	7.1
Rented	135	41.7
Other (private or stay with relative)	166	51.2
Body mass index (BMI)		
18.0-24.99	61	18.8
25.0-29.99	128	39.5
≥30.0	135	41.7
No. of days of emotional ill-feeling		
None	1	0.3
<15 days	228	70.4
>15 days	95	29.3

*1 Emalangeni (E)=US$ 0.12 (E 3,001=US$ 356)

### Distribution of responses to nutritional knowledge, attitude, and practice-related questions

The mean score of nutritional knowledge was 67% (8/12 points) (Table 2). Out of 324 respondents, only 58% (n=190) correctly said ‘false’ to the statement “high-fibre diet is dangerous for HIV-infected individuals”; 24% (n=79) correctly responded that the statement “antioxidants are dangerous for PLWHA” is false; and 67% (n=216) correctly identified the statement “eating vegetables prevents HIV” as false (Table 3). The mean score for nutritional attitude was 67% (24/36 points) (Table 2). As shown in Table 3, 4% (n=12) strongly disagreed that preparing a balanced meal is time-consuming, 19% (n=60) strongly agreed that self-view of nutritional status is important, and 48% (n=155) strongly agreed that eating a variety of foods is key to balanced nutrition. Thirty-nine percent (n=127) reported their source of nutritional information to be family, friends, media, and health professionals.

Concerning hygiene, 24% (n=79) of the respondents strongly disagreed that hygiene is more important than nutrition. Only 30% (n=100) of the respondents reported to have consumed more than four food-groups the previous day, and 66% (n=213) reported eating three meals per day. About 61% (n=246) of the respondents reported that they were exclusively breastfeeding and intended to continue for at least six months. Only 16% (n=53) reported drinking milk always, 31% (n=102) stated that they always included animal protein when cooking, and almost half [47% (n=152)] indicated drinking at least 1 litre of water daily. A proportion of the respondents reported to have eaten the following foods four times in the previous week: grains (6%; n=18), dairy products (12%; n=39), eggs (22%; n=72), animal-based protein (27%; n=86), vegetables (43%; n=139), and fruits (41%; n=133).

### Association of and correlation between sociodemographic and KAP variables

Bivariate analysis showed that the level of education (p=0.003), type of transport (p=0.044), and BMI (p=0.002) were significantly associated with nutritional knowledge. Health facility (p=0.001), religion (p=0.001), principal wage-earner (p=0.031), and emotional status (p=0.030) were significantly associated with nutritional attitude. Educational level (p=0.002), monthly income (p=0.008), religion (p=0.007), employment status (p=0.009), transport used when going for shopping (p=0.001), type of accommodation (p=0.006), and BMI (p=0.015) were significantly associated with nutritional practices. Significant positive correlations were observed between nutritional K and A (r=0.155, p=0.005), nutritional K and P (r=0.456, p=0.001), and nutritional A and P (r=0.230, p=0.001) (Table 5). Monthly income (r=0.177, p=0.001) significantly correlated with nutritional practices positively. A negative significant correlation was observed between the number of people in a household and nutritional practices (r=-0.124, p=0.027).

### Multiple regressions for predictors of nutritional knowledge, attitude, and practices

BMI (p=0.001) and educational level (p=0.001) were predictors of nutritional knowledge while educational level was the only predictor of nutritional attitude. The findings indicated that type of transport used when going for shopping, level of education (p=0.001), employment status (p=0.001), monthly income (p=0.038), knowledge of food proportion in a plate for a balanced meal (p=0.001), knowledge that high-fibre diet is dangerous for individuals on ART (p=0.004), and knowledge that eating a variety of foods is key to balanced nutrition (p=0.006) were significant predictors of nutritional practices (p=0.002).

**Table 2.** Summary of nutritional knowledge, attitude, and practice scores obtained by respondents

Questionnaire	Total score	Minimum score	Maximum score	Mean±SD
Nutritional knowledge	12	1	11	8±2.1
Nutritional attitude	36	12	36	24±3.8
Nutritional practices	41	6	35	21±6.5

**Table 3.** Distribution of nutritional knowledge-related responses of pregnant and lactating women living with HIV in Manzini region (N=324)

Variable	True		Don't know		False	
	n	%	n	%	n	%
Maize is an energy-giving food	310	95.3	11	3.4	3	0.9
Eggs are rich in energy	141	43.5	40	12.3	143	44.1
Carbohydrates and fats are energy-giving foods	261	80.6	45	13.9	18	5.6
Fish is a good source of protein	281	86.7	31	9.6	12	3.7
Fruits and vegetables are rich in vitamins and minerals	285	88.0	24	7.4	15	4.6
Nutrients cannot be provided by just one kind of food	242	74.7	49	15.1	33	10.2
Protein-rich foods are needed to build and repair body tissues	251	77.5	58	17.9	15	4.6
High-fibre diet is dangerous for people on ART	55	17.0	79	24.4	190	58.6
Eating vegetables prevents HIV	66	20.4	42	13.0	216	66.7
HIV infection is a result of poor nutrition	42	13.0	45	13.9	237	73.1
Antioxidants are poisonous for PLWHA	118	36.4	127	39.2	79	24.4
Water is a nutrient	23	7.1	60	18.5	241	74.4

## DISCUSSION

Several studies have indicated socioeconomic status as a major confounder of good nutritional practices (17,18) but there are limited data on how the sociodemographic factors could influence the nutritional knowledge, attitude, and practices among the vulnerable population, such as PLWHA. The purpose of this study was to investigate nutritional KAP and the influence of sociodemographic factors among pregnant and lactating women living with HIV in the Manzini region of Swaziland. The mean age (27±6.1 years) of the pregnant and lactating women living with HIV interviewed in this study fell within the 15-49 years range, who reportedly had the highest prevalence of HIV in Swaziland (3). Out of 324 respondents, 11% were aged below 20 years, a proportion that is half of those in the findings from SDHS (2007), which indicated teenagers (both HIV-negative and positive); they comprised 23% of ANC clients (2). These data could alternatively suggest that 50% of the teenagers attending ANC are HIV-positive, thus indicating a potential vulnerability of this particular age-group.

Although 40% of the respondents reported they were employed or self-employed, only 5% earned a monthly income exceeding 3,000 Emalangeni (US$ 359), indicating that a majority of the respondents were engaged in low-paying jobs. This could be attributed to the low level of education as shown in the proportion (8%) of the respondents who had acquired tertiary education and, hence, inability of the majority to get high-paying jobs. Further indications of low socioeconomic status of the respondents were observed in the low proportion that indicated using cars when going for shopping (8%) and who own houses with mortgage (7%). Our study revealed unsatisfactory mean scores of nutritional knowledge (67%), attitude (67%), and practices (51%). These findings are comparatively lower than the scores observed in women of childbearing age living with HIV in Uganda, who had nutritional knowledge and attitude scores of 88% and 75% respectively (19). The difference could be attributed to the training of the Ugandan women on the importance of nutrition as reported by 89.5% of the women. In Swaziland, little is known on the impact of the nutrition counselling during prenatal and postpartum periods. The present study showed significant positive correlations between nutritional KAP among pregnant and lactating women. Similarly, Petrie *et al*. (20) reported that knowledge on mother-to-child HIV transmission and attitude towards breastfeeding significantly and positively correlated with feeding practices among HIV-infected women in Western Cape.

**Table 4.** Distribution of nutritional attitude-related responses of pregnant and lactating women living with HIV in Manzini region (N=324)

Variable	Strongly disagree		Disagree		Agree		Strongly agree	
	n	%	n	%	n	%	n	%
Preparing a balanced meal is time-consuming	12	3.7	58	17.9	113	34.0	141	43.5
It's important for mothers to know about preparing a balanced meal	26	8.0	7	2.2	77	23.8	214	66.0
It's not vital to eat a balanced meal if already on ART	22	6.8	17	5.2	56	17.3	229	70.7
A nutritious meal can come from one's own small garden	63	19.4	12	3.7	135	41.7	114	35.2
I should eat fruits only when I feel like	17	5.2	98	30.2	59	18.2	150	46.3
Vegetables must be over-cooked to kill microbes	24	7.4	21	6.5	119	36.7	160	49.4
Self-view of nutritional status is important	85	26.2	31	9.6	148	45.7	60	18.5
Hygiene is more important than food and nutrition	79	24.4	147	45.4	61	18.8	37	11.4
Taking supplements is better than eating food	15	4.6	26	8.0	111	34.3	172	53.1
Processed foods are generally better than raw foods	18	5.6	28	8.6	136	42.0	141	43.5
It is not easy to maintain good nutrition for a poor family	92	28.4	74	22.8	77	23.8	81	25.0
Eating a variety of foods in moderation is key to balanced nutrition	64	19.8	32	9.9	73	22.5	155	47.8

As hypothesized in our study, these observations confirm that nutritional knowledge and attitude are important determinants of nutritional practices. Almost half (49%) of the respondents strongly disagreed that “vegetables must be overcooked to kill microbes” while 51% strongly agreed. This raises concern on the nutrition losses during food preparation by the respondents. Thus, there is a need to create awareness on desired food-handling methods that balance between retention of vital nutrients and assurance of food safety. More than half of the respondents (58%) stated that a “high-fibre diet is dangerous for individuals on ART.” This suggests that most respondents were not aware of the various food-groups and their associated health effects. Majority of the respondents (70%) had never heard about antioxidants, indicating that they were unlikely to consume foods rich in antioxidants that react potentially with damaging oxidizing agents in human body. The findings of this study agree with those reported in the Swaziland Non-communicable Diseases Risk Factors Surveillance report (21) that highlighted an overall low consumption of fruits and vegetables among adults. Apart from the nutrition counselling during ANC and postnatal care, community focus group discussions could improve nutritional perceptions and practices among pregnant and lactating women (17).

In the present study, the level of education, type of transport, and BMI were significantly associated with nutritional knowledge whereas the health facility, religion, principal wage-earner, and emotional status were significantly associated with nutritional attitude. The role of religion in influencing the beliefs regarding diet during childhood illness has been highlighted in India (22). The educational level, monthly income, religion, employment status, type of transport used when going for shopping, type of accommodation, and BMI were significantly associated with nutritional practices. A study in Belgium and another study in Australia reported significant associations of gender, age, level of education, and employment status with nutritional knowledge (15,23).

**Table 5.** Pearson's correlation between nutritional KAP and sociodemographic variables in pregnant and lactating women living with HIV in Manzini region (N=324)

	Nutritional knowledge	Nutritional attitude	Nutritional practices	Age	No. of children	No. of pregnancies	People in household	Income	BMI	HIV period	ART period
Nutritional knowledge	1										
Nutritional attitude	0.155**	1									
Nutritional practices	0.456**	0.230**	1								
Age	0.064	−0.004	0.036	1							
No. of children	−0.021	−0.003	−0.042	0.635**	1						
No. of pregnancies	0.003	0.011	−0.027	0.719**	0.889**	1					
People in household	0.049	0.007	−0.124*	0.087	0.279**	0.225**	1				
Income	0.071	−0.027	0.177**	0.197**	−0.033	0.007	−0.087	1			
BMI	0.096	0.038	0.062	0.124*	0.075	0.124*	0.041	0.009	1		
HIV period	0.019	0.021	−0.064	0.308**	0.314**	0.312**	0.181**	0.037	0.032	1	
ART period	−0.006	0.003	−0.029	−0.130*	−0.111*	−0.109	−0.007	0.031	−0.051	−0.075	1

*p<0.05;

**p<0.01;

ART=Antiretroviral treatment; BMI=Body mass index; HIV=Human immunodeficiency virus

To the best of our knowledge, this is the first study on predictors of nutritional KAP for pregnant and lactating women living with HIV in Swaziland. Most of the significant predictors belonged to the construct of nutritional knowledge and sociodemographic factors and included: knowledge of food proportion, employment status, level of education, type of transport used when going for shopping, knowledge that high-fibre diet is not dangerous for PLWHA, knowledge that eating a variety of foods is the key to balanced nutrition, and monthly income. Inadequate formal and nutrition education of the mothers has been identified as important basic causal factor of child malnutrition in Swaziland (24). There is need to consider an inclusive nutrition education programme within the formal education structures, the communities, and the primary healthcare system, especially with more focus on the vulnerable segments of the population, such as pregnant and lactating women living with HIV.

In this study, knowledge of proportion of food in a plate that makes a balanced diet was revealed as significant predictor of nutritional practices. In Swaziland, the concept of eating a variety of foods needs to be emphasized in the various communities through programmes, such as kitchen gardening to enhance accessibility to fruits and vegetables. The surplus produce may be sold by the pregnant and lactating women to generate income and enhance their socioeconomic status. Only 47.8% of the respondents strongly agreed that eating a variety of foods in moderation is the key to balanced nutrition. This particular nutritional knowledge variable was a significant predictor of nutritional practices and is a pointer to the important role of nutrition education when planning nutrition care interventions for pregnant and lactating women. However, as observed in Gambian women by Mwangome *et al.* (25), nutritional knowledge may not obviously translate into nutritional practice; hence, there is need to consider the broader social, cultural and economic factors, including the value of involving men. Findings in New Delhi, India, reported a low translation of nutritional knowledge to practices (26). Therefore, there is need to incorporate sociodemographic variables and continuous interventions that aim at switching nutritional knowledge to good nutritional practices.

### Limitations

Causal directions between KAP could not be established in this cross-sectional study because such an approach would require a longitudinal study design. The lack of similar studies hindered further comparison of the results with other regions or different groups in the country; however, this study forms the basis upon which comparisons with other studies may be made. Face-to-face interviews may lead to socially-desirable responses; nevertheless, interviewers were asked to use the same translation.

### Conclusions

The present study showed that sociodemographics and nutrition education are important determinants of nutritional practices among pregnant and lactating women living with HIV in Swaziland. The study emphasizes the importance of considering sociodemographic factors during policy formulation and planning for and implementation of nutrition care programmes, especially those aimed at pregnant and lactating women living with HIV in Swaziland.

## ACKNOWLEDGEMENTS

The authors appreciate Taipei Medical University (TMU) for offering an assistantship to help the first author to study Masters degree from which the manuscript was written after the Masters thesis. Much appreciation is due to the support from Swaziland National Nutrition Council. Thanks to all the interviewers and participants. It is also an honour to win the First Prize in Nutrition & Biotechnology Group in TMU 2012 Teachers and Students Joint Academic Symposium as an “Excellent Research Paper.”

## References

[1] Joint United Nations Programme on HIV/AIDS. (2011). World AIDS day report 2011. How to get to zero: faster. Smarter. Better.

[2] Central Statistical Office. (2008). Swaziland demographic and health survey: 2006-07.

[3] Swaziland. (2010). Ministry of Health. 12th round of national HIV sero-surveillance in pregnant women attending antenatal care (ANC) services at heath facilities in Swaziland.

[4] Semba RD, Tang AM (1999). Micronutrients and the pathogenesis of human immunodeficiency virus infection. Br J Nutr.

[5] WHO/FAO/UN. (2009). Participant's manual. Nutritional care and support for people living with HIV/AIDS: a training course.

[6] Jiamton S, Pepin J, Suttent R, Filteau S, Mahakkanukrauh B, Hanshaoworakul W (2003). A randomized trial of the impact of multiple micronutrient supplementation on mortality among HIV-infected individuals living in Bangkok. AIDS.

[7] Fawzi WW, Msamanga GI, Hunter D, Renjifo B, Antelman G, Bang H (2002). Randomized trial of vitamin supplements in relation to transmission of HIV-1 through breastfeeding and early child mortality. AIDS.

[8] Fawzi WW, Msamanga GI, Spiegelman D, Wei R, Kapiga S, Villamor E (2004). A randomized trial of multivitamin supplements and HIV disease progression and mortality. N Engl J Med.

[9] Fawzi W, Msamanga G, Spiegelman D, Hunter DJ (2005). Studies of vitamins and minerals and HIV transmission and disease progression. J Nutr.

[10] Kaiser JD, Campa AM, Ondercin JP, Leoung GS, Pless RF, Baum MK (2006). Micronutrient supplementation increases CD4 count in HIV-infected individuals on highly active antiretroviral therapy: a prospective, double-blinded, placebo-controlled trial. J Acquir Immune Defic Syndr.

[11] Fawzi WW, Msamanga GI, Kupka R, Spiegelman D, Villamor E, Mugusi F (2007). Multivitamin supplementation improves hematologic status in HIV-infected women and their children in Tanzania. Am J Clin Nutr.

[12] Austin J, Singhal N, Voigt R, Smaill F, Gill MJ, Walmsley S (2006). CTN 091/CRIT Cartenoids Study Group. A community randomized controlled clinical trial of mixed carotenoids and micronutrient supplementation of patients with acquired immunodeficiency syndrome. Eur J Clin Nutr.

[13] Komwa MK, Jacobsen KH, Parker DC (2010). HIV/AIDS-associated beliefs and practices relating to diet and work in southeastern Uganda. J Health Popul Nutr.

[14] Whaling MA, Luginaah I, Reid G, Hekmat S, Thind A, Mwanga J (2012). Perceptions about probiotic yogurt for health and nutrition in the context of HIV/AIDS in Mwanza, Tanzania. J Health Popul Nutr.

[15] De Vriendt T, Matthys C, Verbeke W, Pynaert I, De Henauw S (2009). Determinants of nutrition knowledge in young and middle-aged Belgian women and the association with their dietary behaviour. Appetite.

[16] McLeod ER, Campbell KJ, Hesketh KD (2011). Nutrition knowledge: a mediator between socioeconomic position and diet quality in Australian first-time mothers. J Am Diet Assoc.

[17] Dykes F, Lhussier M, Bangash S, Zaman M, Lowe N (2012). Exploring and optimising maternal and infant nutrition in North West Pakistan. Midwifery.

[18] Nabhani-Zeidan M, Naja F, Nasreddine L (2011). Dietary intake and nutrition-related knowledge in a sample of Lebanese adolescents of contrasting socioeconomic status. Food Nutr Bull.

[19] Bukusuba J, Kikafunda JK, Whitehead RG (2010). Nutritional knowledge, attitudes, and practices of women living with HIV in eastern Uganda. J Health Popul Nutr.

[20] Petrie KE, Schmidt SD, Schwarz CE, Koornhof HE, Marais D (2007). Knowledge, attitudes and practices of women regarding the prevention of mother-to-child transmission (PMTCT) programme at the vanguard community health centre, western cape–a pilot study. South Afr J Clin Nutr.

[21] World Health Organization (2009). Swaziland non-communicable disease risk factors surveillance report.

[22] Benakappa AD, Shivamurthy P (2012). Beliefs regarding diet during childhood illness. Indian J Community Med.

[23] Hendrie GA, Coveney J, Cox D (2008). Exploring nutrition knowledge and the demographic variation in knowledge levels in an Australian community sample. Public Health Nutr.

[24] Masuku-Maseko SKS, Owaga EE (2012). Child malnutrition and mortality in Swaziland: situational analysis of the immediate, underlying and basic causes. Afr J Food Agric Nutr Dev.

[25] Mwangome M, Prentice A, Plugge E, Nweneka C (2010). Determinants of appropriate child health and nutrition practices among women in rural Gambia. J Health Popul Nutr.

[26] Anand D, Puri S (2013). Nutritional knowledge, attitude, and practices among HIV-positive individuals in India. J Health Popul Nutr.

